# The Silver Jubilee (2025) of Insulin Glargine: Introducing the Era of Long‐Acting Insulin Analogues for Diabetes Mellitus

**DOI:** 10.1111/dom.70751

**Published:** 2026-04-27

**Authors:** Geremia B. Bolli, Philip D. Home, Mauro Lepore, Matthew C. Riddle, Francesca Porcellati, Carmine G. Fanelli, Paola Lucidi, Hannele Yki‐Järvinen, Reinhard H. Becker, David R. Owens

**Affiliations:** ^1^ Department of Medicine and Surgery Perugia University Medical School Perugia Italy; ^2^ Translational and Clinical Research Institute Newcastle University Newcastle upon Tyne UK; ^3^ Tortoreto Ordine Dei Medici Della Provincia di Teramo Teramo Italy; ^4^ Department of Medicine, Division of Endocrinology, Diabetes and Clinical Nutrition Oregon Health & Science University Portland Oregon USA; ^5^ Centro Diabetologia Asl 2 Umbria ‘Centro Storico’ Foligno Italy; ^6^ Helsinki University Hospital Helsinki Finland; ^7^ Minerva Foundation Institute for Medical Research Helsinki Finland; ^8^ Sanofi Diabetes Division Berlin Germany; ^9^ Cardiff University Medical School Cardiff UK

**Keywords:** basal insulin, insulin glargine, insulin NPH, long‐acting insulin analogues

## Abstract

In the year 2000 the first once daily long‐acting bioengineered insulin analogue (LAIA), insulin glargine (‘glargine’), a true basal insulin (BI), became available for clinical use. This led to the decline in the 50‐year‐old era and prominence of the intermediate‐acting insulins, neutral protamine Hagedorn (NPH) and lente, requiring twice daily administration to control the basal metabolism of people with type 1 diabetes (T1DM) and type 2 diabetes (T2DM). This milestone bridged the gap between regimens involving unmodified human insulins of the previous century to those referred to as using ‘designer insulins’, with the introduction 4 years previously of the meal‐time analogue, insulin lispro. The rapid gain in popularity of glargine is explained by its clinical benefits (once‐daily dosing, titration to achieve improved pre‐breakfast plasma glucose, with a lower risk of nocturnal hypoglycaemia compared to NPH, and less frequent blood glucose monitoring). These benefits correlate with the pharmacokinetic/pharmacodynamic characteristics of insulin glargine being closer to physiological BI supply. In T2DM glargine changed the paradigm of insulin substitution by embedding the concept of ‘treating‐to‐target’, by starting BI ‘early’, with focus on near‐normal fasting plasma glucose prior to the introduction of prandial insulin, and more recently in combination with GLP‐1 receptor agonists. These practices/principles have continued with the introduction of additional innovative LAIAs for once‐daily or indeed weekly use. Today glargine remains in widespread worldwide use in people with T1DM and T2DM, is often the initial BI used, while it serves as a reference against which other LAIAs are tested in clinical trials.

## 1 Introduction

The first marketed soluble long‐acting biosynthetic insulin analogue, glargine, was developed by Hoechst (as HOE901), and, after various industrial mergers, introduced as Lantus by Aventis (now Sanofi) after approval by the US FDA and EMEA early in 2000. Market uptake was very rapid, second only to Viagra (Aventis, personal communication). This milestone in the history of use of basal insulin (BI) in insulin‐treated diabetes marked the end of the previous 50 year‐long era of dominance of the intermediate‐acting preparations (NPH and lente). The new era of the long‐acting insulin analogues (LAIAs) begun by glargine 100 U/mL (U100) was quickly enriched by additional candidates (insulin detemir [‘detemir’] and later others). Glargine U100 (and others) was added to the World Health Organization Essential Medicines List in 2021 [1]. In 2023, it was the 30th most prescribed medication in the United States, with more than 18 million prescriptions for an estimated 4 million people [2]. As of 3 March 2026, glargine appears in 62 400 scientific papers in the literature (Google Scholar).

The silver jubilee of glargine is thus an opportunity to review the history of the development of the extended‐acting insulin preparations since the introduction of animal‐extracted insulin over a 100 years ago. In this narrative, the clinical impact of LAIAs on therapeutic strategies for the management of diabetes will be reviewed, with a particular focus on glargine, as first in field.

## 2 The Long Road From the Early Formulations of Insulin Preparations With Extended Activity

The observation, at the beginning of the insulin era, that subcutaneously‐injected insulin from animal pancreas had a sub‐12‐h duration of action, promoted research to prolong the glucose‐lowering effects of unmodified (regular, soluble) insulin [3, 4, 5]. The motivation was to reduce the number of daily injections, rather than any understanding of the need for BI supply. Attempts to mix insulin with gum arabic solutions, oil suspensions, lecithin emulsions or vasoconstrictor substances met with little or no success due to pain on injection, variability in absorption and/or poor stability [6]. Greater success occurred when insulin was complexed with other proteins [7], or when metal ions were added to the insulin solution [8]. In 1936, Hagedorn reported the combination of insulin with the essentially non‐antigenic protein protamine (extracted from trout sperm), thus reducing its solubility at neutral pH [7]. A stability issue was resolved by Scott and Fisher also in 1936 using surplus protamine with added zinc, producing protamine zinc insulin (PZI) [9]. PZI possessed a very prolonged action, but bioavailability was poor, severe hypoglycaemia occurred erratically, and the preparation was never popular [10].

Krayenbuhl and colleagues, working in Hagedorn's laboratory, defined the optimal proportions of insulin and protamine, with no excess of either, hence ‘isophane’ insulin [11]. This resulted in 1946 in neutral protamine Hagedorn (NPH) insulin, a crystalline suspension, in which zinc, phenol, and m‐cresol play critical roles [11]. NPH was marketed in 1950 replacing the two‐vial system of protamine and buffer, or the separate injection of meal‐time unmodified insulin and PZI. In the 1970s, fixed‐ratio combinations of meal‐time and NPH insulins became available.

Scott and Fisher had noted in 1935 that adding excess zinc ions prolonged the action of insulin [8], an effect largely dependent on the physical state and size of the suspended zinc‐insulin particles [12]. This, and competition between the Nordisk and Novo companies north of Copenhagen, led Hallas‐Møller colleagues to the insulin–zinc suspension series of extended‐acting insulins (semilente [amorphous], ultralente [microcrystalline], and lente [a mixture of these]), marketed from the 1950s [12, 13, 14].

## 3 The Concept of Basal Insulin and the Quest for Physiological Substitution

### 3.1 A History

In the first 50 years of insulin therapy, the complex physiology of the regulatory mechanisms of glucose homeostasis was not known. This includes the pattern of diurnal insulin secretion and the different physiological roles of insulin secreted in the fasted as compared to the fed state, only subsequently termed ‘basal’ and ‘meal‐time’ or ‘prandial/bolus’. Indeed, in those decades it was difficult to measure blood glucose, so dose titration depended on urinary glucose excretion, occurrence of hypoglycaemia, and avoidance of hyperglycaemic states. Also, little was known about the relationship between glycaemic control and long‐term diabetic complications before the late 1970s, resulting in considerable controversy [15].

Research was directed to producing preparations with extended activity primarily to limit daily injection number, providing insulin cover with a mixture of meal‐time and extended‐acting insulin preparations in type 1 diabetes (T1DM), and often twice daily extended‐acting insulin in type 2 diabetes (T2DM). Indeed, these regimens remained quite usual until the end of the century. Exceptions included the once‐daily bovine ultralente‐based regimen promulgated by Turner and colleagues (and the initial basis of the insulin arm in the UK Prospective Diabetes Study) and occasional patients able to tolerate PZI. Growing out of the twice‐daily regimens, fixed mixtures (initially unmodified + NPH) became popular in paediatric practice and for T2DM. As compared to the present era, based on our knowledge of physiological insulin replacement, that time was one of intellectual struggle in trying to match insulin preparations to insulin need [5].

The advent of the application of radioimmunoassay from the late 1970s to insulin [16], and the newly discovered C‐peptide, changed that paradigm, notably to distinguish meal‐time insulin secretion from the BI used to control fasting and late post‐prandial blood glucose levels [17, 18, 19]. Co‐incidentally, the development of continuous subcutaneous insulin infusion (CSII) (see Section 3.4) led logically to the understanding of the separate roles of meal and BI, while the advent of recombinant DNA technology allowed the insulin chemists to design modified insulins with more specific meal and basal pharmacokinetic (PK) properties when injected subcutaneously. These understandings were fundamental to the design from the 1980s of rational regimens of insulin substitution therapy [20], with flexible administration of rapid‐acting insulin preparations at meal‐times and insulin preparations with stable prolonged activity (BI) for the fasting hours (night‐time and late post‐prandial periods), for people with both T1DM [18, 19] and T2DM [21, 22].

All this was complemented by the introduction in the late 1970s of the technique of self‐measurement of blood or plasma glucose, making it possible to titrate individual insulin doses of a meal‐time + basal regimen (pump or injections) [20]. This further allowed the glucose control hypothesis to be formally tested in the Diabetes Control and Complications Trial (DCCT) [23].

### 3.2 The Physiological Role of Basal Insulin Secretion

**FIGURE 1:**
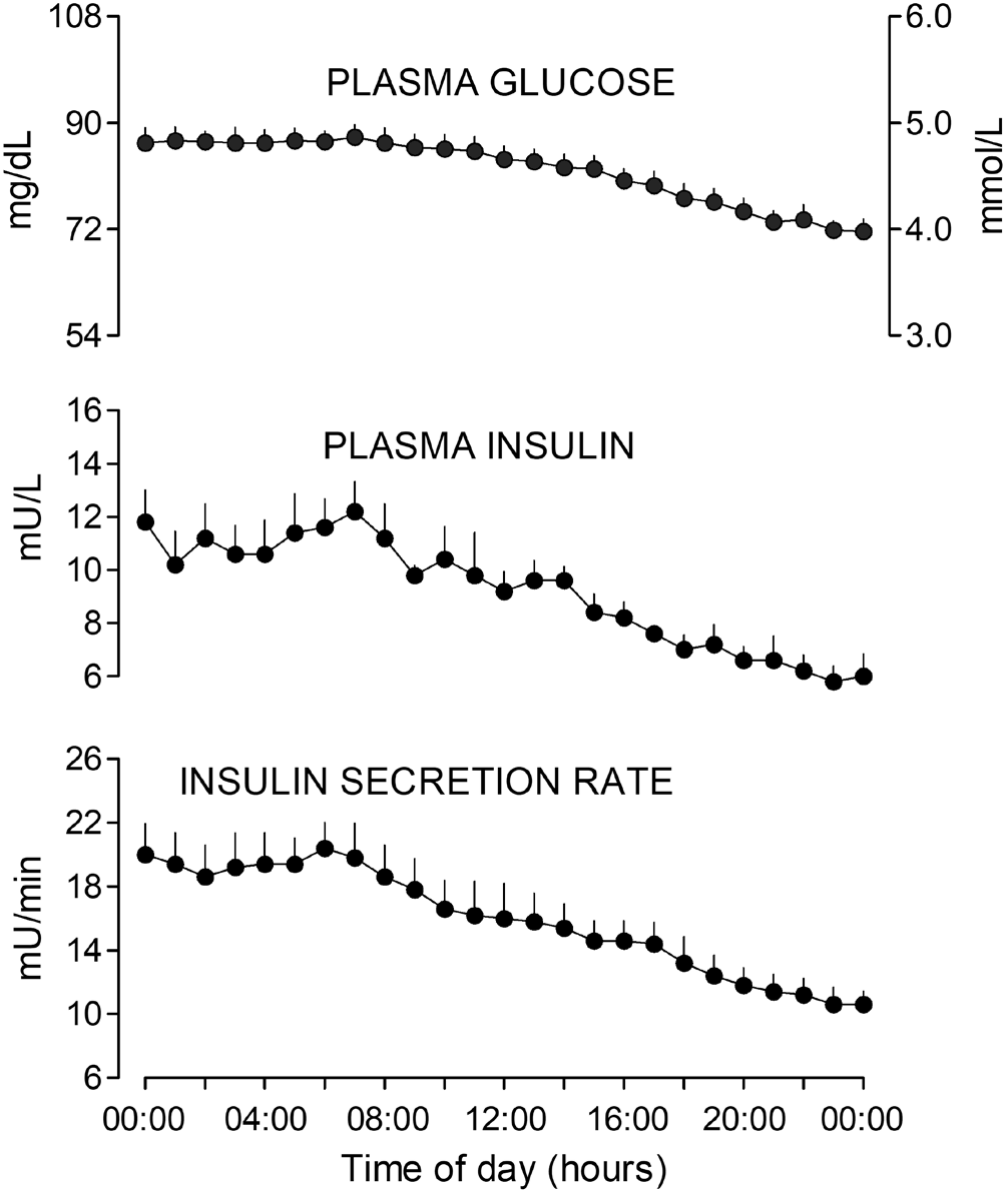
Plasma glucose and insulin concentrations and calculated insulin secretion rate during 24 h of fasting (midnight to midnight) in five people without diabetes (2 males, mean age 29 years, mean BMI 23.6 kg/m^2^). The participants had their last meal 5–6 h before beginning the study (midnight). Note the flat PK of plasma insulin (basal insulin) from midnight to 07:00–08:00 h and the progressive fall thereafter with prolonged fasting. Data are mean ± SE. Unpublished data from pilot experiments performed for the study described in references [25] and [26].

The predominant role of BI is to continuously restrain lipolysis and hepatic glucose output (HGO) in the fasting state, thus preventing excess free fatty acid and glucose concentrations in plasma [24]. Physiologically HGO falls when fasting is prolonged beyond a few hours as substrate supply from liver and muscle glycogen (the latter providing lactate for gluconeogenesis) wanes (Figure 1) [25, 26]. Consequently, the secretion of insulin decreases, lessening the suppression of HGO. With exercise the same happens [27], although here the principle effect of the change (increase) in the portal glucagon: insulin ratio will be to mobilize liver glycogen stores to prevent hypoglycaemia. In people without diabetes the plasma concentration of BI is low, with a nearly flat profile [25, 26], amounting to about 50% of daily insulin secretion [28]. The ideal activity profile of a pharmaceutical long‐acting insulin preparation during fasting and in particular at night should then be to mimic the PK characteristics of the BI of non‐diabetic people (Figure 1), albeit acute changes in insulin requirement (e.g., with exercise) will need decreased insulin supply or action.

### 3.3 The Problematic Replacement of Basal Insulin With the Insoluble NPH and Lente Insulin Classes

**FIGURE 2:**
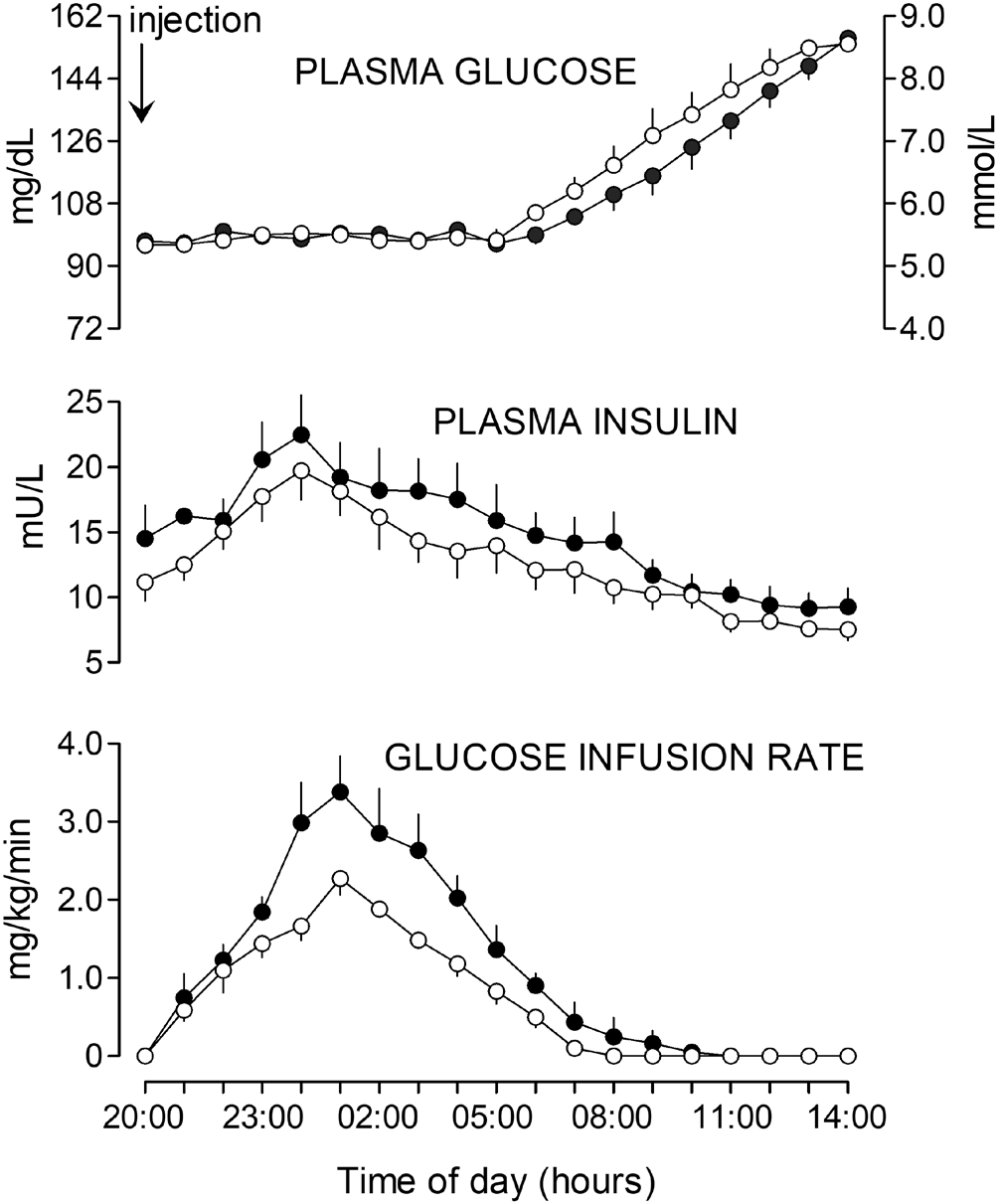
Pharmacokinetics and pharmacodynamics of clinical doses of NPH (0.25 U/kg, full circles) and lente insulin (0.20 U/kg, open circles) injected subcutaneously at 20:00 h in two groups of people with type 1 diabetes on NPH (*n* = 5) or lente (*n* = 5) insulin in the evening, long term. On the study day the participants received human unmodified insulin subcutaneously at breakfast and lunch, and an intravenous feedback insulin infusion from 16:00 to 20:00 h at variable rate to achieve and maintain euglycaemia (dinner was skipped). The studies were done with the non‐automated euglycaemic glucose clamp technique. Data are mean ± SE. Unpublished data from pilot experiments performed for the studies described in reference [34].

Lente and NPH were the only widely used extended‐acting preparations until 2000, and thus had to provide the BI supply overnight and for longer between‐meal intervals. NPH became the more popular as its PK and pharmacodynamic (PD) profile was unaltered when mixed with unmodified insulin [29], and because of compatibility problems of lente with insulin pen‐injectors [30]. In T1DM it was clear that NPH had a duration of action of well under 24 h at usual clinical doses, hence its twice daily use. Further, the glucose‐lowering effect was highly variable from day to day, associated with a considerable incidence of hypoglycaemia due to erratic absorption [31] and the requirement for resuspension of the crystalline suspension prior to injection (10–20 cycles of inversion) [32, 33]. Studies with the glucose‐clamp technique from the 1980s demonstrated the broad peak of activity post‐dosing and confirmed the short profiles (Figure 2) [34]. Although it was possible to prolong the duration of action by increasing insulin dose, that increased the problem of hypoglycaemia. This was an issue in the injection‐treated patients in the intensive arm of the DCCT, where titration of evening NPH to the target FPG resulted in several‐fold increase of the rate of nocturnal episodes of severe hypoglycaemia [23, 35]. For both NPH and lente, the large day‐to‐day as well as within‐day variability of PG proved an independent risk factor for hypoglycaemia [36].

### 3.4 The Insulin Pump Approach to Basal Insulin Supply

After Kadish introduced the concept of continual sampling of blood glucose to adjust the intravenous delivery of an insulin pump [37], work in Germany and Canada, notably on computer‐controlled dosing algorithms, led to the commercialized Biostator essentially for research use [38, 39]. Portable pumps for animal research led to the concept, apparently developed in a London taxi between Keen, Alberti, and Parsons, of open‐loop CSII, with first patient being tried on the system in early 1976, and a patient series published in 1978 [40], and mirrored in 1979 [41]. This finally achieved the goal of constant BI delivery. Optimally titrated CSII resulted in flat and steady PG in the nocturnal fasting hours, in contrast to NPH or lente regimens [42]. With CSII HbA1c was lower compared to NPH and lente regimens [42, 43], with a lower risk of hypoglycaemia [44]. The latter may arise from lower glucose variability with CSII [45, 46], consistent with the earlier PK studies by Binder comparing unmodified and suspension insulin preparations [47].

In 1979 the Oxford group challenged the need for CSII, suggesting the meal‐time + BI regimen of unmodified insulin prandially and bovine ultralente as basal could achieve the same result [48]. However, the poor bioavailability of bovine ultralente prevented its acceptance by others [49], while its human insulin replacement was not a long‐acting insulin. Nevertheless, this established the concept of the meal‐time + BI regimen and set the insulin chemists on a quest leading to true long‐acting insulins. CSII was (and still is) a technologically more complex and expensive option of insulin delivery compared to multiple daily injections (MDI). More recently, the availability of commercial subcutaneous continuous glucose monitoring systems has enabled the wider use of closed‐loop hybrid pump systems, notably in people with T1DM [50].

### 3.5 1996–2000: The Challenge to Basal Insulin Requirement From the Introduction of Rapid‐Acting Insulin Analogues

An unexpected result of the introduction in 1996 of the rapid‐acting insulin analogues to replace meal‐time human unmodified insulin [51, 52], was the changes needed to BI replacement, then with NPH and lente. With unchanged extended‐acting insulin dose or regimen, insulin lispro (‘lispro’) improved the post‐meal glucose levels at 1–2 h, but then allowed a progressive increase of PG compared to unmodified insulin [52]. As a result, lispro, and later insulin aspart, did not result in lower HbA1c [53]. This is a consequence of unmodified insulin contributing late overlap effects to the absorption profiles of NPH or lente. Accordingly, the use of a meal‐time insulin analogue required simultaneous optimization of the extended‐acting insulins to control inter‐prandial and nocturnal PG control. This contrasts with CSII, which effectively reduced HbA1c more with lispro than unmodified insulin [54].

Attempts were therefore made for people with T1DM not on CSII to optimize the replacement of BI supply when using NPH [55, 56, 57, 58, 59, 60]. One approach was based on the concept of dividing the dose into multiple injections [56, 60]. This flattened basal plasma insulin over the 24 h as compared to twice‐daily NPH [60]. However, this four‐times daily NPH regimen was soon overtaken by the introduction of glargine [61].

### 3.6 Designer Long‐Acting Insulin Analogues and NovoSol Basal

The advent of DNA‐recombinant production techniques in the early 1980s opened the possibility of modifying the amino acid sequence of insulin to change the PK of subcutaneous absorption to better mimic the dynamics of physiological secretion. One approach was to synthesize analogues with isoelectric point (pI, the pH of least solubility) close to the neutral pH of the body. Such an insulin analogue can be soluble at the acidic pH of the vial/cartridge but precipitates after the injection in subcutaneous tissue at physiological pH, thus delaying absorption and action of insulin.

Such a candidate long‐acting insulin analogue was OPID 174 (NovoSol Basal), described in the late 1980s [62, 63, 64] and tested in PK and Phase 1 studies in 1989 [65, 66]. Positive charges were added at two points in the insulin B‐chain. NovoSol Basal showed considerable extension of subcutaneous absorption compared to human ultralente (which has a half‐time of around 13 h) in radio‐iodinated‐insulin investigations [65]. However, a small clinical study (*n* = 9, randomized, cross‐over) of 2 weeks duration, with human ultralente as comparator, suggested poorer overnight plasma glucose control, with lower basal plasma insulin concentrations, and other measures suggestive of metabolic decompensation [66]. Further studies in people with T1DM had difficulty in maintaining basal plasma glucose control without marked dose escalation, suggesting over‐slow release and/or poor bioavailability [67]. Further development was suspended in favour of the acylated‐insulin approach, but the concept was pursued for the development of insulin glargine (see Section 4).

Other later approaches to novel LAIAs (acylated insulins and use of large adducts) are addressed in Section 6.

## 4 Insulin Glargine, the First Long‐Acting Insulin Analogue to Reach the Market

### 4.1 Amino Acid Sequence, Prolongation of Absorption, and Metabolism

The development of insulin glargine focused on di‐arginyl (B31Arg, B32Arg) derivatives of human insulin. Di‐arginyl insulin is an intermediate of human insulin maturation in the islet beta‐cell from proinsulin [68]. This insulin is positively charge‐shifted to a pI close to physiological pH and will precipitate in subcutaneous tissue, giving delayed absorption [69]. Although fully active intravenously in humans [70], this di‐arginyl insulin was not as efficacious as NPH insulin when given subcutaneously in dogs [71], and development was discontinued.

Attention then focused on di‐arginyl insulin derivatives, to improve pharmaceutical stability with unchanged receptor binding and pharmacodynamic potency, [69, 72] with substitution at the end of the A‐chain which scarcely affects receptor binding (potency) [69, 71]. Replacement of A21 asparagine with glycine (A21Gly) resulted in a less dense crystal packing, higher water content, reduction in inter‐hexamer interactions, with binding space for a seventh phenol molecule thus improving the retardation principle and avoiding deamidation of A21Asn for enhanced shelf‐life [69, 73]. A21Gly, B31Arg, B32Arg‐human insulin also had less relative IFG‐1 receptor relative binding versus unmodified di‐arginyl insulin [74]. Prolonged blood glucose‐lowering in dogs compared to di‐arginyl insulin [71] promoted the decision to proceed to human studies [71].

After subcutaneous injection this ‘insulin glargine’ immediately precipitates, preventing rapid absorption. Thereafter the amorphous precipitate slowly liberates hexamers with formation of dimers and monomers of glargine. Moreover, the resolubilized glargine is rapidly converted at the site of injection and in the circulation with enzymatic removal of the arginine pair resulting in A21Gly‐human insulin (metabolite 1 [M1]), with lesser further loss of threonine to A21Gly,B30des‐Thr‐human insulin (metabolite 2 [M2]) [75]. Thus glargine, like natural di‐arginyl insulin, is a prohormone which binds to insulin receptors nearly exclusively as its metabolites, mainly M1 [75].

Later in development, as expected, intravenous glargine showed no difference in effects on HGO and peripheral glucose uptake as compared to human insulin [76] confirming longer duration of action arose from changes in absorption kinetics at the subcutaneous injection site.

### 4.2 Pharmacokinetic and Pharmacodynamic Studies

Pre‐clinical studies included those of decreases in plasma glucose in fasting animals, a model of limited statistical sensitivity due to hypoglycaemic counter‐regulation. Despite this, dog data showed double and triple time to peak activity (2.5, 5.0, and 7.5 h, NPH vs. glargine 15 and 80 μg/mL Zn^2+^), and double the duration of action, with consistent plasma insulin profiles.

**FIGURE 3:**
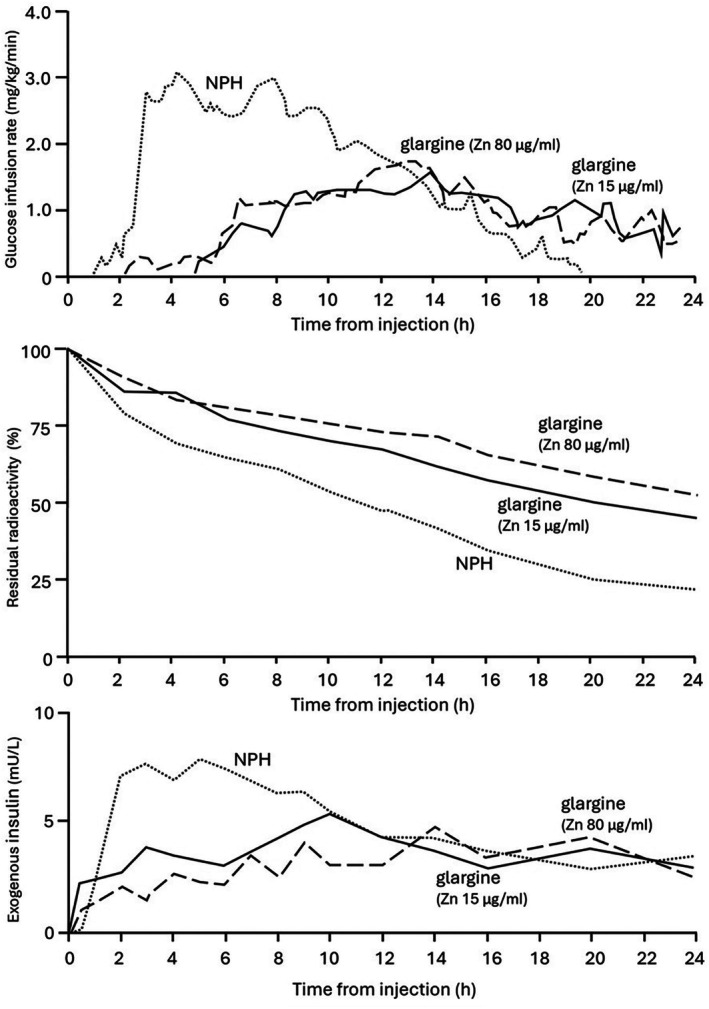
Comparisons of pharmacodynamics (upper panel) and pharmacokinetics (lower two panels) of NPH insulin with insulin analogue glargine (HOE 901) with either 15 or 80 μg/mL Zn^2+^ in non‐diabetic volunteers. Upper panel is median glucose infusion rate after subcutaneous injection of 0.2 U/kg of the insulins studied with the non‐automated euglycaemic clamp technique during somatostatin infusion [77]. Middle panel is injection site residual radioactivity of iodinated insulin and lower panel endogenous‐adjusted plasma insulin concentrations after subcutaneous injection of 0.15 U/kg of the same insulins [78]. Figures redrawn from references [77] and [78]. Insulin glargine is marketed with 30 μg/mL Zn^2+^.

In 1994 the first PK/PD subcutaneous study (double‐blind, crossover) compared glargine (15 and 80 μg/mL Zn^2+^) to NPH in non‐diabetic people [77]. With glargine (both formulations) the maximal activity peaked later (12 ± 4 vs. 6 ± 3 h, mean ± SD) and duration of action was estimated as > 24 h versus 16 ± 2 h as compared to NPH [77] (Figure 3). However, a lower 24‐h area under the glucose infusion curve for glargine compared to NPH (~39% lower with both Zn^+^ formulations) was a concern allowing that some of the action of glargine was evidently beyond 24 h [77]. Further PK studies in non‐diabetic people in 1995 using subcutaneous radiolabelled (^125^I) NPH and glargine found an absorption rate significantly slower with glargine with a T_50_% of 20–24 h compared to 10–12 h for NPH, and a flatter plasma insulin profile [78] (Figure 3).

Also in 1994, the clinical experimental findings in people with T1DM of glargine once daily versus NPH four times daily suggested non‐inferiority [55]. Again, this was of concern—there seemed to be little reason that there would be clinical demand for the new insulin. The company did however decide to continue development beyond the Phase 2 studies, based on clinical opinion that recognized the limitations of NPH in practice, giving a thirst for an insulin with demonstrably novel properties, notably improved duration of action and once daily injection.

PK/PD studies in non‐diabetic people are confounded by continuing secretion of endogenous insulin even if this is partly suppressed by somatostatin or insulin infusion [77, 79]. Clamping for 24 h at the artificial euglycaemia of baseline FPG rather than at the progressively lower plasma glucose of prolonged fasting (Figure 1) [25, 26] is also confounding.

**FIGURE 4:**
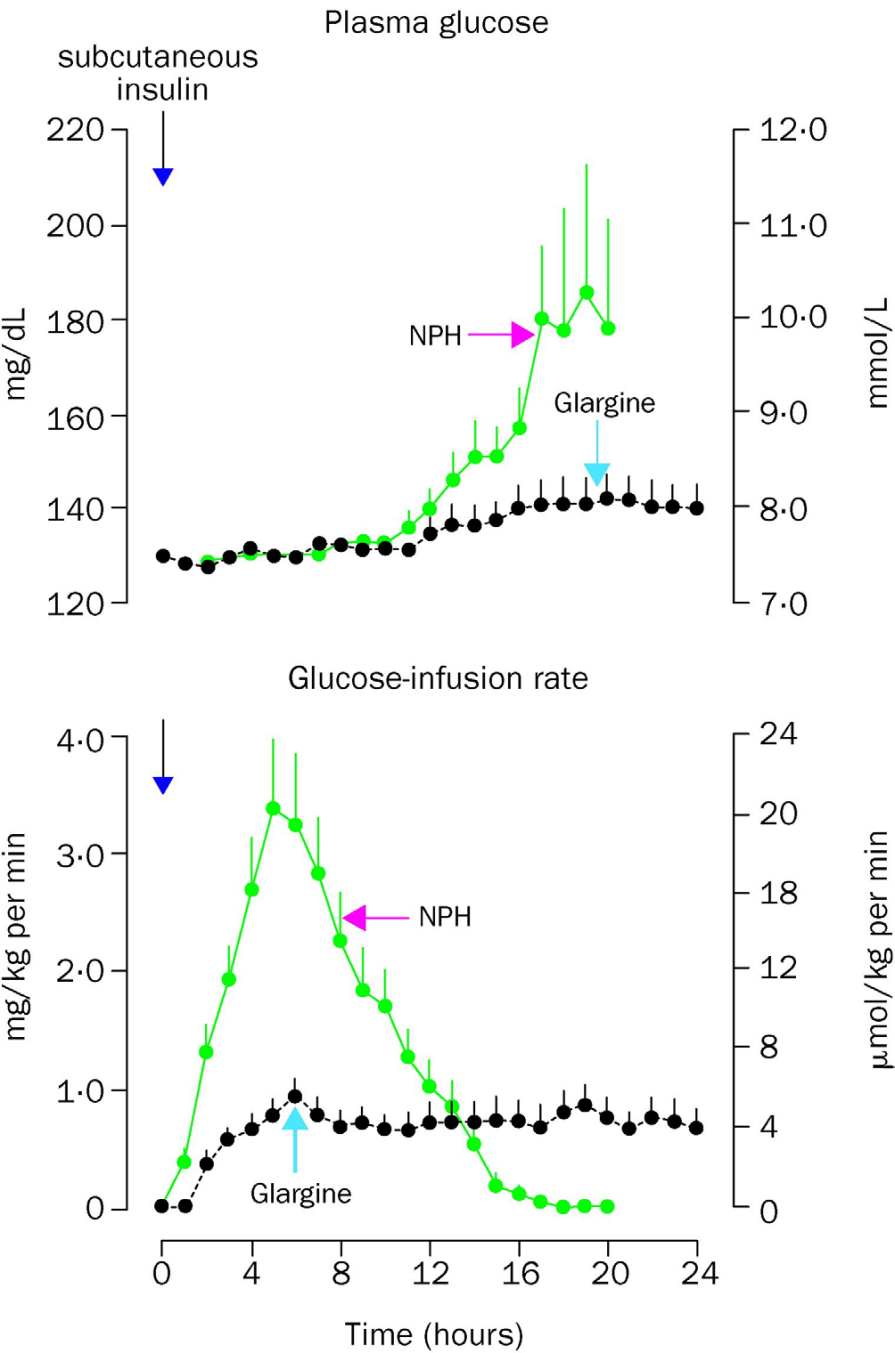
Plasma glucose and glucose infusion rate after subcutaneous injection of 0.3 U/kg insulin NPH or insulin glargine (first injection) in 20 people with T1DM studied with the non‐automated glucose clamp (PG target 130 mg/dL, 7.2 mmol/L). With NPH, all participants terminated the study after 13–20 h because plasma glucose increased > 200 mg/dL (11.1 mmol/L). With glargine, all completed the study to 24 h. Data from reference [80], figure reproduced from reference [81] (with permission).

In 1998 a randomized, double‐blind, cross‐over study of first‐dose subcutaneous glargine versus NPH was performed in people with T1DM using a non‐automated glucose clamp technique [80], at a dose (0.3 U/kg) comparable to the 24‐h dose often used in pump therapy. The clamp target (PG of 7.2 mmol/L [130 mg/dL]) was, however, high to match safe targets for NPH regimens in clinical practice. A distinct peak of action was found for NPH, 5.4 h post‐dosing with end of action at 13 h, while glargine was nearly peakless with end of action at 21 h [80] (Figure 4) [81]. This observation led to postulate that glargine could reduce the risk of nocturnal hypoglycaemia as compared to evening NPH dosing, while delivering lower FPG. Since most people with T1DM did not achieve FPG targets, and nocturnal hypoglycaemia was common, this suggested a large clinical opportunity for glargine, although with the requirement for multiple meal‐time insulin injections.

**FIGURE 5:**
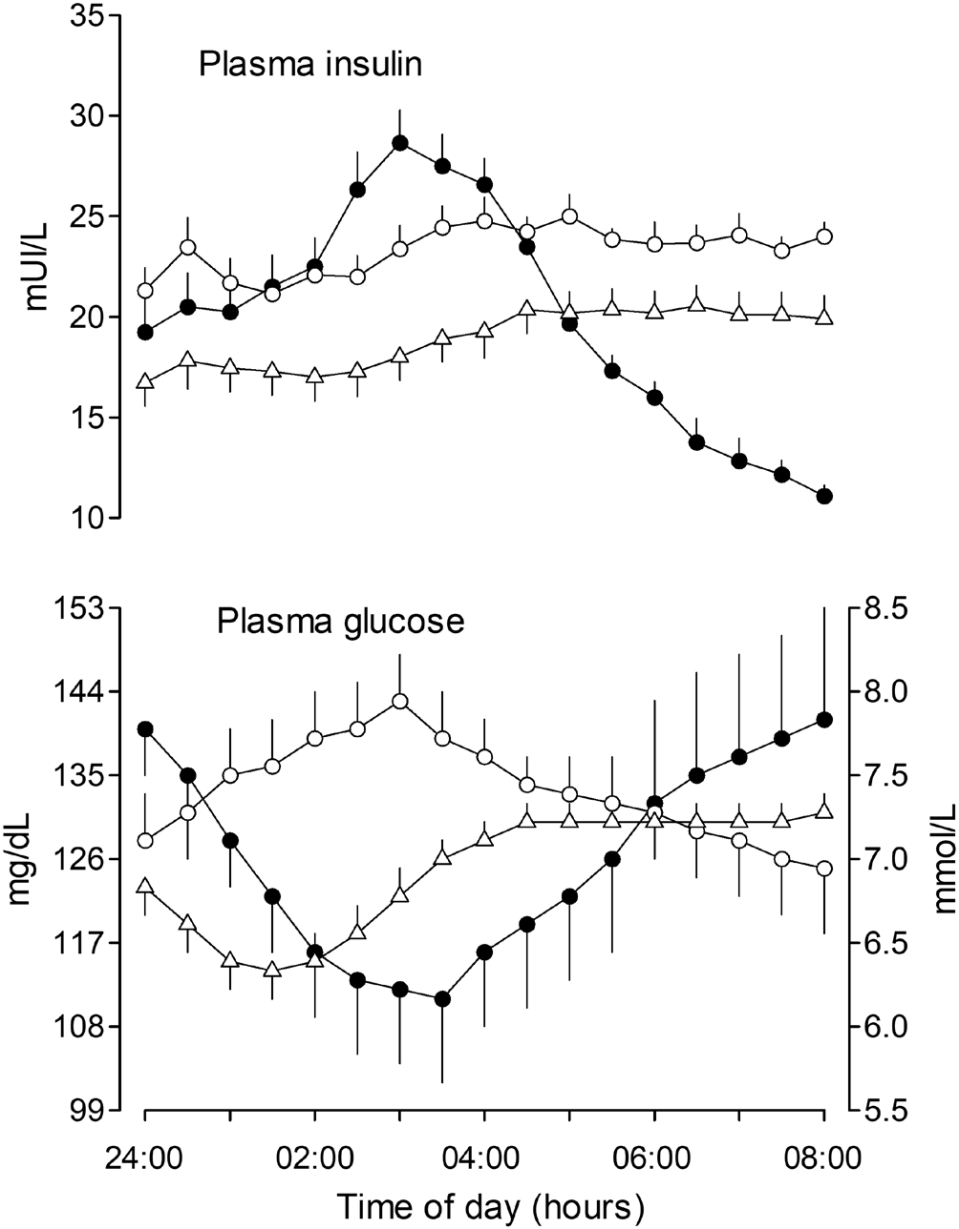
Effects of individual therapeutic doses of insulin glargine, CSII or NPH in 29 people with T1DM on MDI (lispro as prandial insulin). Insulin glargine (open circles, *n* = 10 participants) and CSII (triangles, *n* = 9 participants) show similar nearly peakless nocturnal plasma insulin profiles (thus with lower risk for hypoglycaemia) as compared to the peak effect of bedtime NPH insulin (closed circles, *n* = 10 participants). Data from reference [85].

When the PK/PD of subcutaneous glargine were re‐examined after multiple daily dosing, partly to ameliorate misplaced concerns over ‘insulin stacking’, duration of action appeared even longer (24 vs. 20 h for a single injection) and the 24‐h profile flatter [82]. When the day‐to‐day within‐person variability of PD was studied in non‐diabetic people, it was lower with glargine compared to NPH and ultralente [83]. Further as predicted by the PK/PD study [80], with glargine in an MDI regimen, the so‐called dawn phenomenon observed with NPH and lente in T1DM [84] was ameliorated [85, 86, 87], similar to previous findings with CSII [88]. With glargine as BI blood glucose control was ultimately comparable to that on CSII in preventing nocturnal hypoglycaemia (Figure 5) [85], as well as controlling hyperglycaemia long‐term [45, 89]. This is in contrast with earlier studies of CSII compared with NPH or bovine ultralente in MDI regimens [49, 90].

Early after its introduction, anecdotal reports of late afternoon [91] and/or late evening hyperglycaemia in people with T1DM [92] led to the question of the optimal time of administration of the once‐daily insulin glargine [61, 92] and the possible benefits of twice‐daily administration in selected patients [93, 94]. In a small cross‐over study, twice daily dosing ameliorated the late afternoon rise in plasma glucose [94]. An alternative approach uses a flexible bolus of rapid‐acting insulin analogue (1–4 U) 2–3 h after lunch in addition to once‐daily glargine [93]. Some case series appear to confirm benefit in selected people with T2DM [95]. Such clinical usage has now been usurped by glargine U300 and insulin degludec (see Sections 4.6 and 5.1).

### 4.3 Phase 3 Clinical Trials for Regulatory Approval and Safety Concerns

A series of Phase 3 studies was performed, notably paired studies between North America and Europe in T1DM and T2DM, in the latter for insulin users and the insulin naïve [96, 97, 98, 99, 100]. The aim of these studies was to establish overall efficacy and safety, and indeed HbA1c did not differ from comparator arms using NPH insulin. Marketing authorization does not require superiority, and the evidence of improved glucose control towards the end of the night [101], or reduction in symptomatic hypoglycaemia was not considered hard endpoints. These Phase 3 findings resulted in some consternation in the development and marketing teams of the manufacturer. Fortunately, such concerns were overcome by advisors who saw the clinical attraction of a true once‐daily BI while blood glucose control on current preparations was so poor. Indeed, this is evident from the poor glucose control in those studies, which preceded the treat‐to‐target paradigm. Phase 3 studies are underpowered for hypoglycaemia, but a later meta‐analysis of these and the Phase 4 studies did confirm such an advantage for glargine [102].

Phase 3 studies are also the key pre‐marketing safety studies. In one such study, there was a signal for acceleration of retinopathy [100], and at the time, glargine was known to have a higher growth factor activity than native human insulin, with no understanding as yet that the circulating A21Gly metabolite (M1) [75, 103, 104, 105] has lower relative growth‐promoting activity than human insulin [74]. Accordingly, a specific safety study was designed, confirming no issue in progression of retinopathy levels over 5 years in people with T2DM [106].

A possibly‐related issue arose later (2009), with the publication in *Diabetologia* of a series of observational studies suggesting use of glargine was associated with malignancy [107, 108, 109, 110]. However, a review of patient‐level data from 31 comparator studies in 11 000 people found no increased incidence for any common malignancy [111]. This accords with the circulating glargine metabolite findings (see Section 4.1 and previous paragraph), and, along with the results of the ORIGIN trial [112], put an end to this controversy [113].

Immunological reactions to glargine are rare, such that no difference could be detected in Phase 3 studies compared to NPH insulin. In case reports there is often overlap with reactions to other insulins, which may point to sensitivity to excipients rather than the insulin itself [114].

### 4.4 Phase 4 Studies and the Treat‐to‐Target Paradigm

As expected, glargine was accepted with enthusiasm by the clinical community, even while funders and evidence‐based guidelines were more restrained. As well as being a more pharmacologically appropriate BI in a meal‐time + basal injection regimen, notably in T1DM, the opportunity was taken to build upon the original Oxford suggestion of true BI, rather than pre‐mixes or NPH, as the starter insulin in T2DM [21] and the observation of efficacy of actively self‐titrated evening NPH with metformin [115]. Riddle emphasized that hyperglycaemia in T2DM was predominantly basal [22, 116], that is, the increase in glucose area‐under‐curve over normal was mostly basal from the early morning hours rather than prandial excursions. Further appreciation of the importance of tighter blood glucose control continued to increase, while self‐monitoring technology was becoming more widely adopted. This provided the groundwork for the Treat‐to‐Target Trial of insulin glargine versus NPH once daily using a structured algorithm, whose principal finding was the late night and morning reductions in hypoglycaemia [117]. While HbA1c was not improved in this study as well as in another [118], or in a similar study with insulin detemir (Section 5.1) [119], the inherent simplicity of starting once daily insulin in T2DM took over the world, replacing twice daily approaches, including with pre‐mixtures, eventually even in China and India [120, 121]. The lack of improvement of HbA1c probably partly reflects the amelioration of hypoglycaemia compared to NPH, and partly the study design, namely that dose titration was to the same glucose targets. However, attempts to ‘treat‐to‐hypoglycaemia’ were not successful in showing a difference, perhaps because a longer study would be needed to equalize hypoglycaemia rates [122].

This use of glargine following the Treat‐to‐Target concept was later shown to be efficacious both with user self‐titration versus physician algorithms [123], and when glargine was begun in group sessions as compared to individuals [124].

### 4.5 Insulin Glargine U100 Biosimilars and Copies

The clinical and commercial success of glargine U100 as Lantus made it a prime target for biosimilars (initially termed ‘follow‐on biologics’ in the United States) once patents had expired. Biosimilars follow a regulated pathway of development in most advanced markets and are intended for people starting insulin and for substitution for the originator insulin under medical supervision [125]. More recently, two glargine U100 products have been approved as interchangeable with Lantus (substitution under non‐medical supervision) by the US FDA. In less well‐regulated markets, copies of Lantus have also become available under regulatory processes more akin to those used for generic medicines.

### 4.6 Insulin Glargine 300 U/mL

Observations, in users of high glargine doses, of higher glargine injection concentration (lower volumes) exposed further prolongation of glucose‐lowering effect. With a three‐times higher concentration of glargine (300 U/mL) (Gla300), post‐injection precipitates are denser and more compact, resulting in slower solubilization at the injection site and thus slower absorption into the circulation [126], with a shift in terminal half‐life from 12 to 18 h. The active molecule (mainly metabolite M1) is however the same, and thus has the same receptor binding kinetics (potency) and other chemical properties in the circulation as glargine U100. Associated advances in pen‐technology allowed injection of lower volumes with retained dosage precision. However, Gla300 appears to result in somewhat lower bioavailability after subcutaneous dosing, presumably because of the greater local degradation by tissue proteases during the longer residence time of the precipitate [105]. Accordingly, higher average doses are administered with glargine 300 U/mL to match the glucose‐lowering efficacy of glargine U100 in T1DM [127, 128] and in T2DM [129], as well as those of insulin degludec (‘degludec’) [130, 131].

The more compact subcutaneous depot of Gla300 then explains the flatter and more prolonged PK and PD over the 24 h [132]. The suppression of hepatic glucose production is lower in the first 12 h but greater in the second 12 h post‐dosing, as compared to glargine U100 [128], such that meal‐time insulin doses may need to be lower if pre‐meal PG control is improved. When titrated at bioequivalent doses, Gla300 also shows lower within‐day variability as compared to glargine U100 [128] and degludec (Section 5.1) [131]. The prediction that the flatter PK/PD of Gla300 may be of benefit has been addressed by the clinical trials in T1DM [133, 134, 135] and T2DM [129]. The results show a lower risk for hypoglycaemia at night, and in T1DM a more consistent glycaemic control over the 24 h with attenuation of the pre‐dinner hyperglycaemia (afternoon phenomenon) [91] as compared to glargine U100 [136]. Marketing authorization was granted in 2015.

## 5 Alternative Approaches to Prolonged Action

### 5.1 The Acylated Long‐Acting Insulin Analogues

Insulin detemir (detemir) became the second ‘long‐acting’ BI analogue available for clinical use (approved from 2004). In this molecule the terminal B30Thr was deleted and a fatty‐acid chain attached to the B29Lys residue. Acylation prolongs insulin activity through binding to albumin in the subcutaneous tissue and in the circulation. This bound insulin also decreases variability from fluctuations in absorption [137]. Detemir is unusual in having markedly lower potency in vivo than human insulin [138] and glargine [139] for reasons that remain unclear, despite a pharmaceutical unit of detemir containing four times the molecular concentration of insulin to approximate the same efficacy as human insulin. This effect seems to be more marked in more obese people [140], such that higher detemir unit doses are required for the same glucose‐lowering effect [141]. When the commercial formulation of detemir was compared with the same nominal units of glargine subcutaneously at steady‐state, the biopotency is lower and the duration of action shorter [139, 142]. This suggested a more frequent need for twice daily administration to cover the full 24 h, particularly in people with T1DM and less so in T2DM [141], in line with a terminal half‐life of 6 h, like NPH‐insulin. Detemir, however, compared to the other LAIAs or NPH, offered a small degree of body weight advantage, for reasons still unclear. In the treat‐to‐target study in T2DM, advantage against NPH insulin was shown for hypoglycaemia, but not HbA1c, as for glargine [119]. Head‐to‐head studies of the detemir and glargine were unable to show advantage to detemir, even when twice‐daily dosing was allowed [141]. Detemir is in the process of being withdrawn from some major markets.

Insulin degludec, first approved in 2013, uses a linker to the B29Lys residue to attach a longer fatty acid (hexadecanedioic acid) [143]. After injection the phenol preservative is diluted and diffuses away, causing the modified insulin to form multi‐hexamer chains. As zinc molecules are lost from the ends of these, hexamers and monomers are slowly released. This primary mechanism of prolongation of action, the longest of the daily analogues, is then supplemented by tissue and plasma albumin binding [143]. However, unlike detemir, degludec has full potency in vivo [143].

Studies suggest that degludec results in lower confirmed and symptomatic hypoglycaemia than insulin glargine U100 [144]. However, when tested in a large outcome study, no advantage was shown for cardiovascular endpoints [145]. Both this insulin and glargine U300 have flat enough profiles to be taken (consistently) any time of day, and indeed to allow for some variation (greater with degludec) around the usual time of injection [146, 147]. The formulation also allows the availability of fixed‐ratio combinations with insulin aspart and with liraglutide.

Recently, Novo Nordisk has extended this technology to create a once‐weekly BI. Insulin icodec (’icodec’) utilizes a 20‐carbon fatty acid, again attached to the B29Lys residue, via a longer linker [148]. This results in an insulin with very tight albumin‐binding properties, which, together with some novel amino‐acid substitutions designed to reduce receptor interaction and thus uptake/degradation, extend the glucose‐lowering action to a half‐life of 8 days (estimated on total, not free insulin), not dissimilar to albumin itself [149]. This half‐time cannot provide a constant insulin supply over the week, even after multiple weekly dosing, and, probably as a result, hypoglycaemia is increased compared to comparators (glargine 100 U/degludec) notably on days 2–3 after injection [150]. While approved in Europe, a near doubling of hypoglycaemia in T1DM has proved problematic to the US FDA. The absolute increase in hypoglycaemia in T2DM is however small (because the baseline rate on glargine 100/degludec is low), and approval in T2DM was granted in March 2026. Gan & Lee announced in the same month completion of phase 3 trials of a very similar insulin; details are awaited. The attraction of starting insulin only once weekly, compared to once daily, seems real [151], but at present the advantages and disadvantages over daily BI are not clearly established.

### 5.2 The Abandoned PEGylated Insulin Lispro

A further approach to prolong insulin action was pegylation of lispro with attachment of a 20 kDa polyethylene glycol (PEG) molecule, at B28Lys via a linker [152]. The constraint on subcutaneous absorption rate was molecular size; the PEG chain also protecting the insulin from injection‐site proteolytic degradation. The clinical development programme suggested a long and stable profile of action with less nocturnal hypoglycaemia than glargine U100 [153], while skeletal muscle and adipose tissue activity was reduced, enhancing lipolysis and resulting in relative hepato‐specificity [154]. However, in a modern obesogenic environment, this promotes hepatic fat deposition with elevation of serum alanine aminotransferase levels and VLDL/triglyceride production, with decreased HDL cholesterol [155]. These are all cardiovascular risk factors. Further daytime hypoglycaemia increased compared to glargine U100, presumably secondary to hepatic insulin action countering rescue glycolysis [156]. Development was discontinued.

### 5.3 IgFc Adducts of Insulin—Insulin Efsitora Alfa (Basal Insulin Fc)

Immunoglobulin G2 Fc adducts have been developed by Lilly to prolong action of GLP‐1 receptor agonists (GLP‐1RAs) and insulin, after subcutaneous injection. The size of the fusion protein prevents conventional absorption, while the Fc fragment grants further protection via the physiological FcRN recycling system [157]. Fc adducts also have low antigenicity. PK studies suggest a half‐time in the circulation of around 17 days, and therefore suitable for weekly administration, with a peak‐trough ratio of around 1.1 over the days of the week [158]. The downside of such duration of action is complexity in dose titration. In T2DM compared to glargine U100 in Phase 3 studies there was no difference in HbA1c or hypoglycaemia in insulin naïve or prior MDI users [157, 159, 160]. A small increase in hypoglycaemia of around 20% with a signal for increase in severe hypoglycaemia was found in T1DM, with degludec as comparator [161]. At the time of writing the insulin is submitted for marketing approval.

## 6 Advances in Therapeutic Management From the Long‐Acting Insulin Analogues

### 6.1 Comment

Twenty‐five years of the era of the LAIAs from the introduction of glargine in 2000 have consistently and irreversibly changed the scenario and the paradigm of insulin replacement therapy in general and BI in particular as compared to the previous NPH era. NPH (and the zinc‐insulin series) were introduced to overcome the problem of the short‐acting nature of unmodified insulin, while the BI concept in T2DM can be traced to the Oxford group's promotion of bovine ultralente to manage FPG in the late 1970s [21]. The latter was found unusable by most other clinicians due to dosing problems, though the concept of dose titration by pre‐breakfast plasma glucose estimation was shortly to be revolutionized by patient‐friendly self‐measurement systems [162]. So it was only with glargine (and to a lesser extent detemir) that LAIAs were able to displace NPH for people with diabetes, both in T1DM with a lower risk of nocturnal hypoglycaemia for similar or better overnight glycaemic control in insulin regimens with either meal‐time unmodified human insulin [98] or lispro [86, 87], and in T2DM additionally with better quality of life and easier insulin starts with single daily dosing [101]. The series has evolved, notably with glargine U300 and degludec, while EMA approval in 2024 of the first weekly BI analogue icodec has opened additional options [163].

The lower risk for hypoglycaemia with glargine and the other LAIAs, due to their more physiological PK/PD, has encouraged the harnessing of improved self‐measurement to adopt more ambitious glycaemic targets, for the first time demonstrated in the milestone ‘Treat‐to‐Target’ trial [117]. While comparative HbA1c was not improved, the acceptable and achieved standard dropped markedly, as seen by comparison of this and the equivalent detemir study [119] with the Phase 3 glargine studies. These newer goals had been far from reality in the NPH era, largely because of push back of dosing from the risk of hypoglycaemia [117, 118, 119]. Of note, optimal BI substitution with LAIAs has allowed better understanding of the need to improve prandial glycaemic control, usually after adequate titration of BI dose, and established the modern paradigm in T2DM of replacing BI first and only later managing prandial hyperglycaemia [24]. However, the GLP‐1RAs, often now started before insulin is considered, and which offer advantage in combination with any LAIA as either separated dosing or fixed‐ratio mixtures [164], have provided further useful clinical options [24].

After 25 years no practicing clinician would any longer challenge the efficacy, the safety and the indication of use of glargine and the other LAIAs to replace the BI needs in T1DM and T2DM. It is unfortunate that pricing of NPH insulin still means it is preferred over the advantageous LAIAs in some markets, even in the era of biosimilars of glargine U100. From NPH to glargine was > 50 years, but the pace of change has also notably accelerated after the introduction of recombinant DNA technology derived insulins with glargine's introduction in 2000 clearly marking a new era.

### 6.2 The Future

Glargine U100 may remain the most prescribed BI for the time being, and the prime target for biosimilars [124], but the introduction of further LAIAs emphasizes that the problems of subcutaneous administration of BI, although usefully ameliorated, have not been eliminated [3, 5]. Injections remain an issue to some users, and while pumps introduce cosmetic and technological issues to some, weekly insulins have still to show they can overcome their PK/PD (and thus hypoglycaemia) limitations. As practised today, subcutaneous insulin administration absorption still suffers variability due to site of injection and insulin dosage, compounded by variability in insulin sensitivity due to changes in human behaviour, so hypoglycaemia will continue to be a significant challenge and limit insulin dosing schedules. Ideas for glucose‐sensitive insulins have made slow but encouraging progress [165], but do not approach usability. However, feedback insulin delivery from closed loop systems (using fast‐acting insulin for basal control) has made tremendous progress, but is technology dependent, costly, and still unable to eliminate all risk of hyperglycaemia [166, 167].

## Author Contributions

G.B.B. wrote the first draft. All other authors have equally contributed to rewriting and editing the text. The final text was approved by all. However, M.C.R. contributed to the drafting of this review, but his death prevented approval of the final manuscript. G.B.B. is the guarantor of this work, has full access to the new data in the review, and takes responsibility for the integrity of the analysis thereof and its accuracy.

## Funding

This article was funded in part by the University of Perugia and did not receive any additional funding from the public, commercial, or not‐for‐profit sectors.

## Conflicts of Interest

G.B.B. has no dualities of interest. P.D.H. has received funding from all the major insulin and biosimilar manufacturers, including recently Sanofi and Eli Lilly. M.L. has no duality of interest. F.P. receives clinical trial funding, advisory board and lecture fees from Abbott, AstraZeneca, Lilly, Novo Nordisk, Sanofi. C.G.F. and P.L. have no dualities of interest. H.Y.‐J. has received consulting fees from Novartis, NovoNordisk, Eli Lilly; has participated on a data safety monitoring boards or advisory boards for MSD, Eli Lilly, Hanmi. R.H.B. is a retired employee of Hoechst/Sanofi. D.R.O. has no dualities of interest.

## Data Availability

Data sharing not applicable to this article as no datasets were generated or analysed during the current study.
